# The “Hypertension Approaches in the Elderly: a Lifestyle study” multicenter, randomized trial (HAEL Study): rationale and methodological protocol

**DOI:** 10.1186/s12889-019-6970-3

**Published:** 2019-05-29

**Authors:** Daniel Umpierre, Lucas Porto Santos, Cíntia Ehlers Botton, Eurico Nestor Wilhelm, Lucas Helal, Gustavo Zaccaria Schaun, Gustavo Dias Ferreira, Angélica Trevisan De Nardi, Lucinéia Orsolin Pfeifer, Anderson Donelli da Silveira, Carisi Anne Polanczyk, Graciele Ferreira Mendes, Hirofumi Tanaka, Leonardo Alves, Leony Galliano, Linda S. Pescatello, Maria Laura Brizio, Patrícia Martins Bock, Paula Campelo, Ruy Silveira Moraes, Marlos Rodrigues Domingues, Beatriz D. Schaan, Cristine Lima Alberton, Stephanie Santana Pinto, The HAEL Study Group, Elisa Portella, Héctor Ferreira, Larissa X. N. da Silva, Nórton L. Oliveira, Raíssa Monteiro

**Affiliations:** 1 Department of Public Health, Universidade Federal do Rio Grande do Sul, Porto Alegre, RS Brazil; 2 National Institute of Science and Technology for Health Technology Assessment (IATS/HCPA), Hospital de Clínicas de Porto Alegre, Clinical Research Center, Rua Ramiro Barcelos 2350, Porto Alegre, RS Brazil; 3 Exercise Pathophysiology Laboratory, Graduate Program in Cardiology and Cardiovascular Sciences, Universidade Federal do Rio Grande do Sul, Porto Alegre, RS Brazil; 4 Department of Sports, Universidade Federal de Pelotas, Pelotas, RS Brazil; 5 Department of Physiology and Pharmacology, Universidade Federal de Pelotas, Pelotas, RS Brazil; 6 Cardiology Division, Hospital de Clínicas de Porto Alegre, Porto Alegre, RS Brazil; 7 Medical School, Universidade Federal do Rio Grande do Sul, Porto Alegre, RS Brazil; 8 Department of Kinesiology and Health Education, The University of Texas at Austin, Austin, TX 78712 USA; 9 Department of Kinesiology, University of Connecticut, Storrs, CT 06269-1110 USA; 10 Faculdades Integradas de Taquara, Taquara, RS Brazil

**Keywords:** Older, Aged, Aging, Exercise, Physical activity, Clinical trial

## Abstract

**Background:**

Hypertension is a clinical condition highly prevalent in the elderly, imposing great risks to cardiovascular diseases and loss of quality of life. Current guidelines emphasize the importance of nonpharmacological strategies as a first-line approach to lower blood pressure. Exercise is an efficient lifestyle tool that can benefit a myriad of health-related outcomes, including blood pressure control, in older adults. We herein report the protocol of the HAEL Study, which aims to evaluate the efficacy of a pragmatic combined exercise training compared with a health education program on ambulatory blood pressure and other health-related outcomes in older individuals.

**Methods:**

Randomized, single-blinded, multicenter, two-arm, parallel, superiority trial.

A total of 184 subjects (92/center), ≥60 years of age, with no recent history of cardiovascular events, will be randomized on a 1:1 ratio to 12-week interventions consisting either of a combined exercise (aerobic and strength) training, three times per week, or an active-control group receiving health education intervention, once a week. Ambulatory (primary outcome) and office blood pressures, cardiorespiratory fitness and endothelial function, together with quality of life, functional fitness and autonomic control will be measured in before and after intervention.

**Discussion:**

Our conceptual hypothesis is that combined training intervention will reduce ambulatory blood pressure in comparison with health education group. Using a superiority framework, analysis plan prespecifies an intention-to-treat approach, per protocol criteria, subgroups analysis, and handling of missing data. The trial is recruiting since September 2017. Finally, this study was designed to adhere to data sharing practices.

**Trial registration:**

NCT03264443. Registered on 29 August, 2017.

**Electronic supplementary material:**

The online version of this article (10.1186/s12889-019-6970-3) contains supplementary material, which is available to authorized users.

## Backgrounds

Hypertension is a leading risk factor for disability-adjusted life years globally [1] and contributes to chronic diseases with low quality of life and high mortality rates. Advanced age and elevated blood pressure (BP) exponentially increase mortality risk, which underscore the importance of BP control in the older population [2]. Among nonpharmacological interventions, structured exercise programs are strongly recommended for adults with elevated BP or essential hypertension [3]. In older subjects, exercise interventions are cornerstone due to multiple effects that may benefit not only vascular disease markers [4] but also other outcomes related to physical function [5, 6].

Meta-analyses of several controlled intervention studies have generated estimates that, individually, aerobic or resistance exercise training can reduce systolic BP levels by 6 to 8 mmHg in patients with hypertension [7, 8]. However, two main aspects limit extended evidence extrapolation. First, trials have mostly used either aerobic or resistance exercise training alone in young or middle-aged individuals without hypertension. Second, given that the combination of aerobic and resistance training has become a recommended mode of exercise training, it is relevant to investigate the efficacy of combined training in older individuals with hypertension. Although combined exercise programs can enhance the nonpharmacological treatment of hypertension in the elderly, such sample has been scarcely addressed in clinical trials [9, 10]. Moreover, inferences are considerably constrained by only a minority of studies examining BP as a primary outcome together with a high risk of bias indicated by lower-to-median scores of methodological quality [9].

We aim to evaluate the efficacy of a combined aerobic and resistance exercise training program on reducing BP levels compared with an attention control group undergoing health education in older patients with hypertension (≥ 60 years old). Herein, we will describe the HAEL Study, which is a parallel, randomized (1:1 allocation ratio), controlled by active intervention, blinded for outcome assessors and data analysts, multicenter, superiority trial, using ambulatory BP as the primary outcome. The choice of the active comparator group aims to reach a principle of equipoise and partially account for research participation effects (Hawthorne effect). Our main hypothesis is that the exercise training program will lead to greater reduction in systolic ambulatory BP in comparison to the control group. Secondary outcomes related to cardiovascular, mental, and physical function were chosen due to their relevance for the elderly. Based on the design of interventions and variables of interest, we hypothesize that the combined exercise training program will lead to superior changes in secondary outcomes when compared to the health education.

## Methods

### Study setting

This multicenter trial takes place in Porto Alegre and Pelotas, cities located in southern Brazil. Porto Alegre is the coordinator center, at the Hospital de Clínicas de Porto Alegre, and centralizes most of the methodological procedures discussed below. The research teams for each center have similar sizes and identical structures of investigator roles (Additional file 1). The equipment and space used for interventions at both centers are similar and will be detailed in the *Interventions* topic. The present study protocol follows as closely as possible the SPIRIT Statement 2013 [11]. The World Health Organization Trial Registration Dataset is provided herein (Additional file 2).

### Eligibility criteria

Inclusion and exclusion criteria for subjects are defined as follows. Study centers were chosen by convenience and no eligibility criteria was defined a priori for care-providers:

#### Inclusion criteria

1. Diagnosis of hypertension as assessed by a previous ambulatory BP monitoring (no later than six months) or current use of anti-hypertensive drugs.
2. Age ≥ 60 years old.
3. Unchanged pharmacological scheme for four weeks prior enrollment.
4. Willingness to participate in either intervention group.

#### Exclusion criteria

1. Inability or unwillingness to give informed consent for participation.
2. Myocardial infarction, revascularization procedures, deep vein thrombosis, cerebrovascular events or pulmonary embolism within the last 12 months.
3. Presence of chronic heart failure with NYHA classes III or IV or unstable arrhythmia.
4. Presence of chronic lung disease requiring use of corticosteroid or oxygen therapies.
5. Consumption of more than 14 alcoholic drinks per week.
6. Presence of kidney disease requiring dialysis.
7. Language, hearing or cognitive issues limiting communication.
8. Plans to move outside the areas of HAEL study sites during the period of participation.
9. A friend or relative living in the same household is a study participant.
10. Presence of progressive neurological disorders (Parkinson’s disease, multiple sclerosis, etc.)
11. Cancer requiring treatment within the past two years.
12. Medical report indicating moderate or high risk for exercise-related event [12], based on the initial maximal exercise test and clinical evaluation.

### Interventions

The HAEL participants are randomly allocated either to a combined exercise training program or to a health education intervention, each lasting 12 weeks. Detailed description on both interventions is provided below:

#### Combined exercise training

In Porto Alegre, exercise sessions take place at a communitarian exercise facility external to the teaching hospital. In Pelotas, exercise sessions take place at an exercise facility within the School of Physical Education, Universidade Federal de Pelotas.

Supervised exercise sessions lasting approximately 60 min, 3 days per week. The session consists of an initial warm-up (< 5 min), followed by 20–30 min of aerobic exercise in moderate intensity, 4–5 exercises, 2–3 sets of resistance training (lasting from 15 to 20 min), and 5–10 min of cool-down. The intensity of walking/running is based on the original Borg rating at 12–14 of perceived exertion [13], whereas resistance exercises are based on OMNI rating of 4–8 (out of 10) of perceived exertion scale [14]. Prescribed movements are identical for both centers and consist of multi-joint resistance exercises emphasizing major muscle groups and daily-life activities like sitting, standing up, pushing and pulling. To achieve greater external validity and applicability in environments with limited resources, the exercises are based on bodyweight and elastic band resistance, which require low complexity for setting up, are affordable and can be performed in limited space. The last 5–10 min of each session serve as the cool-down period, during which subjects perform stretching and mobilization exercises. During this time, the exercise supervisor addresses one of hypertension-related topics, based on the same contents planned to the health education group, however, with a brief informative approach lasting 2 to 5 min. Progression for resistance training sessions is based on more intense and faster contraction speed (Table 1). BP measurements are carried out before every exercise session to ensure that subjects BP are below 180 and 100 mmHg for systolic and diastolic BP, respectively. These pre-exercise BP values are documented once a week in subject’s records.

**Table 1:** Resistance training prescription within the combined exercise program

Resistance training variable	Initial prescription (weeks)	Progression (weeks)
Number of sets	2 (1–3)	3 (4–12)
Intensity^a^	Light to moderate (1–3)	Moderate to high (4–12)
	Target: 4 to 6, out of 10	Target: 6 to 8, out of 10
Number of exercises	4 (1–6)	5 (7–12)
Contraction speed^b^	Moderate (1–6)	High (7–12)

^a^assessed by OMNI rating of perceived exertion scale. ^b^concentric contraction performed as fast as possible

#### Health education

In Porto Alegre, this intervention takes place at the Center of Clinical Research at the Hospital de Clinicas de Porto Alegre. In Pelotas, the intervention takes place at the School of Physical Education, Universidade Federal de Pelotas. The intervention consists on educational program with weekly lectures of approximately 60 min of duration. Each lecture is led by health professionals who follow a content script unified for both centers. By using expositive and interactive approaches, topics cover basic knowledge related to hypertension and therapeutic management. Table 2 shows all topics addressed in the health education group. Before the weekly education sessions, participants’ BP levels are measured and documented.

**Table 2:** Topics covered in the health education intervention

Topics in health education for hypertension	
1.Getting to know hypertension	
2. Hypertension and risk	
3. Signs, symptoms and urgencies	
4. General treatment for hypertension	
5. Medication and adherence	
6. Diet-sodium intake	
7. DASH diet	
8. Alcohol and tobacco	
9. Psychological stress	
10. Weight loss and risk reduction	
11. Physical activity	
12. Wrap-up and celebration	

*DASH:* Dietary Approaches to Stop Hypertension

#### Hypertension management

**Fig. 1:**
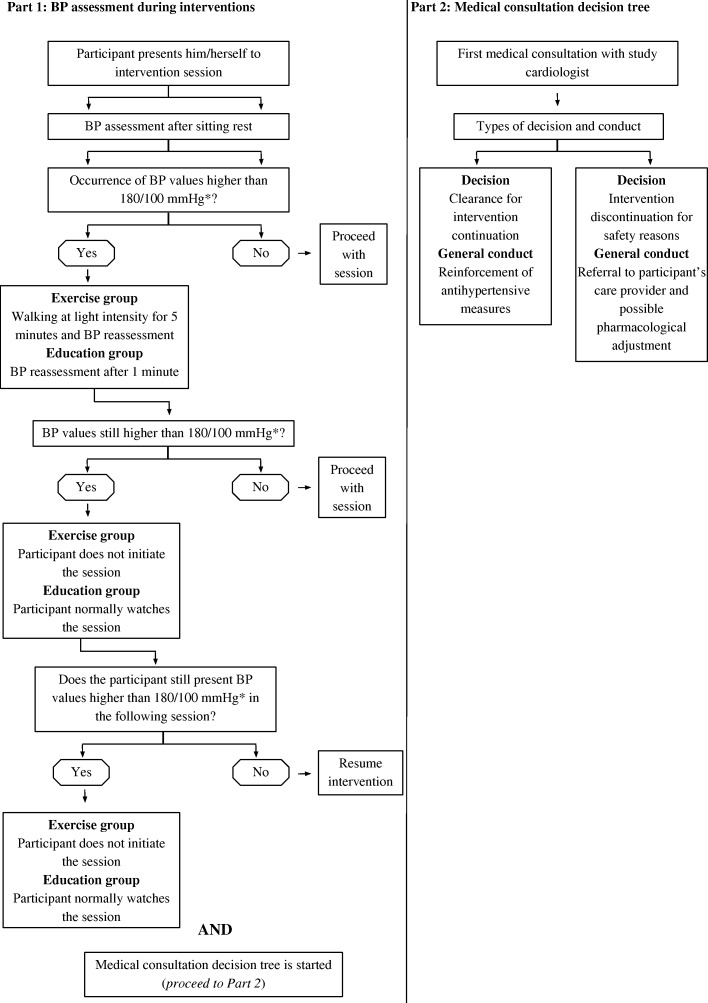
Decision tree algorithim for incidence of excessively high resting blood pressure values before intervention sessions. * indicates values of systolic and diastolic blood pressure, respectively

For safety reasons, some criteria are implemented to manage participants with uncompensated BP along the study (Fig. 1). Participants allocated to any intervention arm presenting sustained pre-session systolic or diastolic BP equals to or greater than 180 mmHg or 100 mmHg, respectively, at two subsequent sessions must undergo a medical appointment. Such values are defined by at least two measurements per session for participants in both groups. In addition, participants in combined exercise training are invited to walk for 5 min at light intensity to reduce a possible anticipatory BP elevation prior the exercise; in these cases, measurements occur after due rest after such short walk episode.

Therefore, participants with elevated BP according to the cutoff values mentioned above should be examined within seven days through consultation carried out by participants’ physicians or study cardiologists. In consultations provided by study cardiologists, an algorithm will be followed (Fig. 1). In brief, whenever a consultation occurs, participants should receive medical clearance to continue to participate. In appointments conducted by study cardiologists, no pharmacological adjustment will be made at a first appointment for participants receiving medical clearance. Therefore, such consultations will be based on reinforcing measures of antihypertensive management. Whenever a second medical appointment is necessary, pharmacological adjustments will be considered to ensure BP control and risk reduction. Such cases may fulfill criteria for discontinuation and will only be kept in interventional under medical recommendation.

#### Criteria for discontinuing allocated interventions

A participant may be discontinued from the study at the investigator’s discretion for safety reasons. For subjects allocated to any group, an incident cardiovascular event, hospitalization or severe health event during the intervention period are considered criteria to discontinue study participation. Examples of a severe health event may include: sustained uncompensated BP for 2–3 times during the protocol (systolic BP ≥ 180 mmHg or diastolic BP ≥ 100 mmHg, respectively), and medical illness that precludes attendance to intervention sessions. In addition, muscular or joint injuries (e.g., muscular, joint) impairing the participant to follow the intervention are considered exclusion criteria for participants allocated to combined exercise training.

#### Strategies for trial retention

During weekends, participants allocated to both groups receive text messages with Institutional Review Board (IRB)-approved content to reinforce time and place of intervention sessions. For the health education group, the message content is based on the topic that will be covered at the next class, whereas the exercise training group receives four slightly different messages along each month of intervention (three monthly cycles of four messages). We use phone calls to inquire for any adverse events if a participant misses a session of any intervention arm. The phone calls schedule is ceased for participants declaring their withdrawal from the study.

### Outcomes

In each center, both randomized groups are assessed for the outcomes listed below by standardized methodological procedures and a similar schedule (Table 3). Outcomes are measured for all randomized participants, irrespective of attendance or completion status. For participants who drop out of the study at any time after the randomization, research personnel use contact information to invite such individuals to undergo the end-study outcome assessments (12 weeks after intervention onset).

**Table 3:** Time scheme for study conduction

	Study period							
	Enrolment	Baseline measures	Allocation and run-in^*^	Post-allocation			Close out	
TIMEPOINT**➔**	t-1	t0	t1	t2	t3	t4^†^	t5^†^	t5^†^
*Timepoint description*	Interviews	Occurs in 2 or 3 evaluation visits	–	Intervention Start	Evaluation visit (6th week)	Intervention end	Final evaluation visit 1	Final evaluation visit 2
ENROLMENT:								
Eligibility screening	x							
Informed consent	x							
Allocation			x					
INTERVENTIONS								
Combined exercise program				x	x	x		
Health education program				x	x	x		
ASSESSMENTS								
*Primary outcome*								
Ambulatory blood pressure		x					x	
*Main secondary outcomes*								
Office blood pressure		x					x	
Endothelial function^‡^		x			x		x	
Cardiorespiratory fitness^§^		x						x
*Other outcomes*								x
6-min walking test distance		x						x
SPPB		x						x
Quality of life questionnaires		x					x	
Autonomic function^‡^		x					x	
*Additional measurements*								
Anthropometric measurements		x					x	
Medication adherence scale (MMAS-8 Questionnaires)		x					x	
Blood variables collection		x					x	
Self-reported physical activity		x			x		x	
Grip strength		x			x			x

*SPPB* Short physical performance battery; *MMAS* Morisky Medication Adherence Scale. * = run-in period will be no longer than 2 weeks. † = time between t4 and t5 will be no longer than 2 weeks. ‡ = analysis of blood pressure and heart rate variability, exclusive for data collected at the coordinator center; § = cardiorespiratory fitness assessment occurs in a third separate visit at the participating center. Use of the©MMAS is protected by US copyright and registered trademark laws. Permission for use is required. A license agreement is available from Donald E. Morisky, 294 Lindura Court, Las Vegas, NV 89138–4632; dmorisky@gmail.com

#### Primary outcome

The primary study outcome is systolic BP assessed by 24 h ambulatory BP monitoring measured before and after three months of intervention. Systolic BP was chosen as the primary outcome due to its linear rise in relation to age and powerful prediction of cardiovascular events in older adults [15, 16]. The study timeframe was chosen to allow an adequate time range to effects (if any) take place, while optimizing trial logistics and participants adherence to interventions [7].

The ambulatory BP values will be treated as individual values for diurnal (from device placement to 10 PM, within the evaluation day), nocturnal (from reported sleep time to reported waking time) and 24 h periods, aggregated as group means at baseline and 12 weeks (trial end). Baseline measurements are carried out no longer than 30 days before the first intervention session whereas post-intervention assessment occurs within 10 days after the last session.

#### Main secondary outcomes

A set of secondary outcomes clinically relevant for the elderly populations was established, including diastolic BP, endothelial function, and cardiorespiratory fitness. Together with systolic BP, the diastolic BP will be assessed through ambulatory and ‘at office’ measurements. Endothelial function is determined by flow-mediated dilatation measured by high resolution ultrasonography at baseline, mid-intervention (6 weeks), and post-intervention (12 weeks), in agreement with published guidelines [17]. Due to resource availability, this outcome is assessed only in participants allocated at the coordinator center. Cardiorespiratory fitness is determined by peak oxygen consumption (VO_2_peak) obtained by maximal cardiopulmonary exercise testing at baseline and 12 weeks. Data regarding both main secondary outcomes will be aggregated as group means at the measurement timepoints.

#### Other outcomes

Other outcomes include complementary measures of physical function, habitual physical activity, adherence to pharmacological therapy, quality of life, and autonomic function. Physical function is evaluated by: (1) total walking distance, as assessed by the six-minute walk test [18], expressed as the longest distance walked at baseline and 12 weeks; (2) lower-limb functional capacity assessed by the Short Physical Performance Battery (SPPB), expressed by scores from 0 (worst performance) to 12 (best performance) based on 3 standing balance tests of increasing difficulty, five sit-to-stand attempts, and a 3-m walk test; and (3) handgrip strength, measured by a hand dynamometer. Self-reported physical activity is measured by the International Physical Activity Questionnaire (IPAQ) [19]. Medication adherence is assessed by Morisky’s 8-item adherence scale (MMAS-8) [20–23]. Quality of life (QoL) outcomes are evaluated by: (1) geriatric depression symptoms, as assessed by the total score on the 15-item geriatric depression symptoms scale (GDS-15) [24, 25]; and (2) scores of QoL, as assessed by the World Health Organization Quality of Life questionnaire (WHOQoL-OLD) [26]. In addition, participants allocated at the coordinator center undergo an autonomic modulation assessment by beat-to-beat BP variability and heart rate variability analysis at rest sympathetic stimulation with the Stroop Color-Word Conflict Test.

#### Safety outcomes

The harms occurring over the period of participation in the trial are defined as adverse events as established by the National Institutes of Aging [27]. Such events are classified according to their severity (mild, moderate, severe), predictability (expected or unexpected), and potential relation with study procedures (definitely related, possibly related, or unrelated). We collect and manage solicited and spontaneously reported adverse events. There is no fixed collection schedule for assessment of harm outcomes, however, spontaneous reports have been documented, communicated to the IRB and managed at demand. In addition, triggers for inspection include absence to one intervention or measurement sessions.

There is no formal adjudication committee, but adverse events are discussed and, if necessary, adjudicated by at least four (50%) out of the following investigators: principal investigator. (D.U), study director (S.S.P), study medical director (B.D.S), study managers (L.P.S, E.N.W, C.E.B), and expert consultants (C.A.P, L.S.P).

### Sample size

Sample size for the primary outcome was calculated using estimates of effect sizes from (i) a meta-analysis of exercise training interventions and (ii) an randomized clinical trial based on a behavioral intervention for middle-aged patients with hypertension, evaluated by ambulatory BP monitoring, and analyzed in an intention-to-treat approach [7, 28]. We estimated that 184 participants would provide power values of 0.79 and 0.92 to detect differences of 2.5 mmHg and 3.0 mmHg between the two group mean values for the 24-h systolic BP, with an expected standard deviation of 6.0 mmHg. Such power calculation comprises: (1) an excess of 22 subjects due to the expected dropout rate (10–15%), (2) intraclass correlation of 0.1 in order to account the proportion that the center-to-center variance may affect the response variance, and (3) a two-sided significance level of 0.050 obtained from a mixed effects model fit without the treatment-by-center interaction.

### Recruitment

The recruitment period for the HAEL Study is planned to range from September 2017 to March 2019. Recruitment phase began from September 2017 to December 2017. During this period, screening questionnaire was administered to 111 individuals (98 of those were not included due to exclusion criteria) and the total enrollment comprised 13 individuals. This was the initial phase to implement the processes for trial conduction in both centers.

From January 2018 on, we have established recruitment targets by center for participant inclusion.

To accomplish such targets, a multifaceted approach is used according to each center resources and comprises screening of electronic medical records as well as advertising means based on recruitment-billboards, newspaper releases, e-flyers in social media, word of mouth, and personal references. In addition, we developed a web site for the HAEL Study (www.ufrgs.br/hael) by which we present study relevant information and contact details. All communication and publicity materials have received IRB approval.

### Assignment of interventions and blinding

Once included in the study, the participant receives an internal number to be de-identified. Sequence of allocation is based on computer-generated random numbers (www.random.org; randomness via atmospheric noise), 1:1 ratio, with permuted blocks of random sizes that are not disclosed to ensure concealment. Allocation concealment is implemented through a central randomization routine conducted by investigators with access to the randomized list (list holders: D. U, S.S.P, C.L.A.) and investigators charged with requesting the code to place subjects to their intervention group. In brief, assigners fill an online request whenever one or more subjects should enter an intervention arm. Thereafter, one of the list holders consult the code in consecutive order and uncover the code relative to the requested subject(s). Such requests are documented and archived for further accountability. To ensure intervention blinding, communication with participants is not carried out by the investigators involved in outcome assessments.

Blinding is implemented for outcome assessors and data analysts (double masking) of primary and secondary outcomes listed in this protocol. Due to the nature of interventions, the study staff conducting or supervising exercise or educational sessions as well as participants are not blinded. To ensure masking of the assessor, subjects are asked to omit their assigned group and not to talk about their interventions during outcome evaluation sessions. In the case of unintentional unblinding due to any reason, it is mandatory for involved researchers to notify the center coordinator. In such cases, participant ID, date, and unblinding circumstance are documented for internal control.

### Data collection

A manual of operating procedures (MOP) was written to increase the consistency for implementation of assessments and interventions across the two study centers. In addition, standard operating procedure (SOP) documents are available for each assessment. Outcome assessors were trained and the handling of a SOP short version is mandatory during each data collection. A data collection committee formed by members of the two centers gathered before the trial onset to consolidate data collection procedures between centers. Periodic meetings and written communication are established to promote internal transparency and consistency.

All variables are assessed at baseline (prior randomization) and at study completion, whereas endothelial function, self-reported physical activity and handgrip strength are additionally assessed at the 6th week of intervention. A participant timeline for the study is presented in Table 3.

#### Measurement of primary outcome

##### Ambulatory BP

In the assessment of 24-h BP, subjects wear an ambulatory BP monitor (90,207, SpaceLabs, Redmond, WA, USA) on the non-dominant arm for 24 h. Participants are asked to refrain from exercise the day prior to and during the recording period. While wearing the monitor they are also asked to maintain a diary of daily activities to record any abnormal activities (such as highly stressful situations, increased physical exertion, etc.) and sleep hours. Data on self-reported sleep times will be used to analyze daytime and nighttime patterns of ambulatory BP monitoring. Ambulatory BP exams are considered valid when at least 70% of the expected readings are available, otherwise, an additional measurement is necessary [29]. Subjects allocated to the combined training intervention undergo the ambulatory BP assessment at least 24 h and no later than 10 days after the last exercise session.

#### Measurements of secondary outcomes

##### Office BP

After sitting the subjects in a calm environment for 5 min, a researcher measures the subject’s BP using calibrated and automated oscillometric devices (OMRON Healthcare Inc., Bannockbur, IL, USA), according to hypertension guidelines [30]. Three measurements, 1–2 min apart, are performed in the arm with the highest initial value. The average of the three measurements is considered the subject’s office BP.

##### Cardiorespiratory fitness

Subjects undergo a maximal cardiopulmonary test on a treadmill. Rates of oxygen uptake, carbon dioxide and volume of expired air are recorded breath-by-breath during an incremental walking/running protocol, with VO_2_peak determination when criteria for test termination is reached [31]. All tests are supervised by a trained exercise physiologist and a physician, using a ramp protocol with rate of increments (both speed and elevation) implemented at their discretion, based on participants’ clinical history, aiming a test lasting from 8 to 12 min. Subjects are asked to take their medications normally and refrain from caffeine consumption prior testing. Due to different gas analyzers between study centers (Porto Alegre: Cortex Metalyzer 3B, Leipzig, Germany; Pelotas: VO2000 MedGraphics, Ann Arbor, MI, USA, respectively), VO_2_peak average values and CI 95% will be checked at the study completion. If a difference is identified between centers, VO_2_peak will be reported accordingly.

##### Walking distance

Subjects undergo a six-minute walking test conducted in a flat 30 m course, in which the total distance walked “as fast as possible” is assessed. Researchers are not allowed to give verbal encouragement other than standardized neutral cues each minute.

##### Lower limbs functional capacity

Subjects complete the SPPB which is a 3-step testing that assesses balance, walking speed, and lower limbs muscular endurance [32, 33]. The balance stage of the test is comprised of three balance challenges of increased difficulty. The walking speed test assesses the 3-m regular-pace walking speed. The muscular endurance assessment involves standing-up five times from a chair as fast as possible without using the arms. The final test score is calculated as a sum of the scores obtained in the three tests. Each test has a maximum score of 4 points.

##### Geriatric depression symptoms

Participants are asked to complete a version of the Geriatric Depression Scale 15 (GDS-15) validated for Brazilian Portuguese [25]. This questionnaire is comprised of 15 questions and is validated to assess depressive symptoms in the Brazilian elderly population. Due the personal nature of the provided information, questionnaires are answered by the participant alone and assisted by researchers only if needed.

##### Quality of life

Participants answer the WHOQOL-OLD questionnaire, translated and validated forBrazilian elderly population [26]. This questionnaire is comprised of24 questions and is an estimate for QoL in 6 different domains. As justified in the procedure above, questionnaires are answered by the participant alone and researchers provide help only if requested.

##### Autonomic function

Autonomic function is assessed by BP and heart rate variability (MP150, Biopac Systems, USA). Phalangeal beat-to-beat BP is recorded (in a sampling rate of 1000 Hz) at supine rest for 10 min and during a 5-min application of a variation of the Stroop Color- Word Conflict test [34]. This test is a mental stress challenge to sympathetically stimulate the subjects. We calculate the BP and heart rate variability based on a spectral analysis of systograms and tachograms. This method provides three frequency band components (Very Low Frequency, Low Frequency and High Frequency) from which autonomous control can be inferred.

##### Endothelial function

To determine the flow-mediated dilatation (FMD), longitudinal images are obtained with the use of high-resolution ultrasonography (HD7XE, Phillips, USA). To do so, a high frequency transducer (3-12 MHz) records the dilatation of the brachial artery for 120 s immediately after the release from a 5-min total occlusion maneuver. The subjects are asked to fast for at least 6 h prior to the procedures. Brachial Analyzer Software (Vascular Tools, Medical Imaging Application, USA) is used to quantify changes in arterial diameter from baseline to post-cuff occlusion. Flow-mediated dilation will be calculated as the percentage change in arterial dimeter fromaverages of 10 baseline diastolic diameters and 3 maximum, systolic diameters post-cuffocclusion. In addition to the pre-and post-trial measurements, a mid-term FMD assessment is conducted during the 6th week of intervention.

#### Control variables

##### Anthropometric assessment

The subject is weighted on a calibrated scale, with light clothes and no shoes. Height is assessed through an analogic stadiometer, during a light inhale and with the head positioned in the Frankfurt plane. Waist circumference is assessed in the midpoint between the iliac crest and the 10th rib.

##### Adherence to pharmacological plan

Subjects answer a validated Brazilian version of the MMAS-8 [20–23], which is comprised of eight self-reported items related specifically to adherence to anti-hypertensive medication scheme. This scale’s score is divided into three categories: high adherence, moderate adherence and low adherence.

##### Blood variables

Blood samples are collected for quantification of total cholesterol, HDL-cholesterol, creatinine, and glycated hemoglobin (HbA1c). In brief, after 12 h of fasting, an experienced technician collects 4 mL of blood from the antecubital fossa. In the coordinator center, blood samples are taken directly to the clinical pathology laboratory (central laboratory) where they are centrifuged and subsequently analyzed. In the participant center, blood samples are centrifuged and stored in a − 80 °C freezer. After 10–20 participants are sampled either at t0 or t5 (Table 3), the samples are transported in a thermic container filled with dry ice to the central laboratory for analysis.

##### Physical activity levels

subjects answer a validated Brazilian version of the IPAQ [35]. The IPAQ long version is used, which assesses physical activity in five independently domains, namely: (a) job-related, (b) transportation, (c) housework, (d) recreational and (e) time spent sitting.

##### Handgrip strength

isometric handgrip strength is measured in both arms with an analogic hand dynamometer (Jamar Sammons Preston Rolyan, Bolingbrook, IL, USA). After one research team member demonstrates proper device and body positioning, the subject keeps an upright standing posture and position his/her evaluated arm with the forearm parallel to the ground (elbow flexed at 90°). Thereafter, the subject is instructed to perform a maximal squeezing contraction with sustained (isometric) effort lasting 5 s. Three attempts are carried out in each arm with one-minute rest intervals.

### Adherence assessments

Measures of adherence to interventions will be reported as group averages and operationalized as attendance and compliance. Attendance is monitored through session’s frequency recording and will be treated as the percent of intervention sessions experienced by a participant given the total number of scheduled sessions (36 sessions for the exercise program or 12 sessions for the education program). Adherence will be treated as the percent of intervention sessions fully accomplished without protocol deviations given the total number of scheduled sessions. For example, this may include either: (i) a participant allocated to the exercise program that, for any reason, walked less than the prescribed duration for a given session; or (ii) a participant allocated to the education program that only partially watched a lecture.

### Data management

At the two study centers, data are collected on standardized paper forms identified by subject number and trial ID and containing instructions for standardized operational procedures. From these forms, we proceed with double data entry for primary, secondary, and additional outcomes. Data entry is carried out at each study center, however, data are centrally stored and managed through the use of REDCap electronic data capture tools hosted at the Hospital de Clínicas de Porto Alegre. REDCap (Research Electronic Data Capture) is a secure, web-based application that will provide us with (1) an intuitive interface for validated data entry; and (2) audit trails for tracking data manipulation and export procedures [36]. Audition for missing or inaccurate data is conducted at the coordinator center. Data are backed up daily by automated export procedures from secure servers of the Hospital de Clínicas de Porto Alegre.

In addition, brachial artery images for endothelial function assessment will be analyzed at an external laboratory (Cardiovascular Aging Research Laboratory, Austin, Texas). To do so, we will share image files over a secure cloud-based sharing platform (Box Inc., USA) hosted by the University of Texas at Austin.

### Statistical considerations

For primary and secondary outcomes, we will adhere to the intention-to-treat (ITT) principle and analyze all randomized participants, irrespective of attrition. The primary hypothesis will be tested on a superiority framework. Variables from ambulatory BP will be treated as diurnal, nocturnal and 24-h for both systolic and diastolic BP. Mixed effects models will be used to determine differences between groups using final values for systolic and diastolic ambulatory BP, adjusted for baseline values (pre-intervention). If these data present low linear fit, we will compare groups using generalized estimating equations with an independence model as the covariance matrix.

Two analysis sets will be established as follows: (1) a full analysis set (FAS) including all randomized subjects, therefore allowing ITT analyses; and (2) a per-protocol (PP) analysis set including all subjects that completed the trial (completers) with adherence to at least 70% of the intervention sessions (≥ 25 sessions for participants allocated in the exercise program, and ≥ eight sessions for participants allocated to the education program). Participants that drop out of the study due to safety concerns or other outcomes will not be censored in PP analysis. Additionally, we plan to carry out a subgroup analysis stratifying both groups by individuals with non-controlled BP before the intervention versus individuals with well-controlled BP before the intervention. Such stratification will be based on tertiles of systolic BP at baseline, with a primary interest in the comparison of interventions on ambulatory BP using the lowest and highest thirds.

Incomplete data will be explored in sensitivity analyses by pattern mixture model. This procedure will describe whether there is an interaction between the main missing patterns and other variables (e.g., group, time, group by time, BP status, BP status by group). We expect two main missing data patterns based on an indicator of completers versus non-completers (defined as randomized subjects without 12-week data). Because withdrawals may occur due to specific harms or other non-anticipated reasons, we will assess whether additional grouping (indicator) should be made due to different patterns of missing data. Therefore, we will qualitatively document reasons and details of withdrawals on a case-by-case basis. In the case of identifiable patterns indicating that missing data are nonignorable, the interpretation of related findings should take missingness into account. No interim analyses other than monitoring of demographic data are planned.

Continuous variables will be summarized according to intervention groups at baseline, if applicable, and end of trial using arithmetic or geometric means, standard deviations, ranges, and interquartile ranges as appropriate. Change from baseline will be summarized descriptively accompanied by its 95% CI. In descriptive summaries, last observation carried forward (LOCF) will be employed to impute missing values. Categorical variables at baseline and end of trial (if applicable) will be summarized as absolute number and proportion of subjects (%) according to intervention groups.

### Monitoring

#### Data monitoring

The HAEL Study does not have a data monitoring committee due to limited resources. We reason this committee would not be mandatory due to the characteristics of interventions and outcomes, despite its highly value for the overall quality of the trial.

#### Harms

The identification, possible solutions, and documentation of adverse events are based on a study management algorithm requested and approved by the Institutional Review Board from the coordinator center.

#### Auditing

If necessary, auditing will be conducted by the Hospital de Clínicas de Porto Alegre through defined protocols implemented by an independent monitoring team adjunct to the IRB structure.

### Ancillary, post-trial care and harm from trial participation

After enrollment in the HAEL Study, each participant receives a brief report of health status prepared by a researcher not involved in intervention implementation and data assessments. If requested, punctual tests information can be given to participants for treatment matters. For harms suffered during trial enrollment related to the study (after adjudication by the committee), we planned contingency actions to assist the participant through care provided either at primary health care units or at the Hospital de Clínicas de Porto Alegre (tertiary care). Finally, the HAEL Study staff has Basic Life Support training and an algorithm for major adverse events is available if necessary.

### Dissemination policy

We aim to disseminate the methods and findings of the HAEL Study to as many stakeholders as possible. Therefore, our dissemination plan after trial completion encompasses the following: (1) breakfast meeting with study participants by which we will present a layman-friendly explanation about the study design, findings, and interpretation; (2) press releases written by journalists and directed towards the general public, and (3) scientific manuscripts. For the latter, criteria for authorship on HAEL Study publications will adhere to the recommendations by the International.

Committee of Medical Journal Editors [37] and those defined by the destination journals. Because we have established a relatively large multi-author group, some publications will carry authorship by a group name designated the HAEL Study Group. When submitting a manuscript authored by HAEL Study Group, byline authors will be mostly defined by full-time equivalents of workload in trial activities. To this end, we do conduct monitoring of all investigators workload on a weekly basis. To ensure reporting completeness, manuscripts written by the HAEL Study Group must adhere to the CONSORT Statement [38], or, if applicable, more suited reporting guidelines.

## Discussion

The HAEL Study presents features which are relevant to be highlighted. First, we established our research question towards the elderly population. Beyond a justification of scantiness of large exercise studies in samples exclusively composed by older individuals, elderlies represent our population of interest primarily because they yield a high prevalence of hypertension and present an exponentially increased risk of death [2]. Therefore, assessing the efficacy of lifestyle interventions that may positively influence BP control in this population is desirable, particularly because older individuals are common polypharmacy users and present reductions in both physical and psychological and/or cognitive domains. Second, we chose to implement a combined training intervention as the candidate method to provide superior effects in BP reduction, vascular adaptation, and functional measures. Importantly, this type of intervention has been recommended in several position stands of exercise to maximize health benefits because both cardiovascular and neuromuscular stimuli occur in parallel. Moreover, we simplified the choice of aerobic and resistance exercises so that the program may be more feasibly implemented in public health settings or low-resource scenarios. Third, we designed the comparator intervention to minimize possible differences due to participation (Hawthorne) effects as well as provide participants with information on varied topics related to hypertension management. Although the interventions differ in weekly frequencies, equating the frequencies of both programs in three times a week would make education meetings more repetitive and probably reduce attrition rates.

Finally, we point out that the HAEL Study is confirmatory trial by nature. Therefore, we have designed this trial establishing methodological standards as high as possible for both outcome measurements and trial management. In this regard, we emphasize some aspects such as the (i) use of 24-h ambulatory systolic and diastolic BP, which is scarcely available from previous trials; (ii) a standardized management plan for participants with uncompensated BP; and (iii) open research practices that will likely make the trial more useful and reproducible.

## Additional files

Additional file 1: Roles of investigators. (DOCX 13 kb)

Additional file 2: World Health Organization Trial Registration Dataset. (DOCX 13 kb)

Additional file 3: Amendments chronology. (DOCX 12 kb)

## Abbreviations

BP: Blood pressure
FMD: Flow-mediated dilatation
IPAQ: International Physical Activity Questionnaire
IRB: Institutional review board
ITT: Intention-to-treat
LOCF: Last observation carried forward
MOP: Manual of operating procedures
QoL: Quality of life
SPPB: Short Physical Performance Battery
VO2peak: Peak oxygen consumption
