# Supplementary material for: The “Hypertension Approaches in the Elderly: a Lifestyle study” multicenter, randomized trial (HAEL Study): rationale and methodological protocol
Source: BMC Public Health. 2019 May 29;19:657. doi: 10.1186/s12889-019-6970-3 (PMC6542055; doi:10.1186/s12889-019-6970-3)
Supplement: Supplementary file 1 — Roles of investigators. (DOCX 13 kb) [file 12889_2019_6970_MOESM1_ESM.docx]

**Additional file 1**

**Roles of investigators**

*Coordinator committee*

Daniel Umpierre, Stephanie S. Pinto, Cristine L. Alberton, Marlos R. Domingues, Beatriz D. Schaan, Cíntia E. Botton, Eurico N. Wilhelm, Lucas Helal, Lucas P. Santos

*Recruitment committee*

Cíntia E. Botton, Eurico N. Wilhelm, Gustavo Z. Schaun, Graciele F. Mendes,Larissa X.N. da Silva, Lucas P. Santos.

*Assessment committee*

Angélica T. De Nardi, Eurico N. Wilhelm, Gustavo Z. Schaun, Gustavo D. Ferreira, Mariana B. Pinto, Laura Milan Vasques, Graciele F. Mendes,Hirofumi Tanaka, Lucas Helal, Lucinéia O. Pfeifer, Patrícia M. Bock

*Intervention committee*

Elisa Portella, Larissa X.N. da Silva, Raíssa Monteiro, Patrícia M. Bock, Maria Laura Brizio, Leony Galliano, Hector Ferreira, Paula Campelo.

*Expert consultants*

Cardiology: Carisi Polanczyk, MD, PhD

Exercise and hypertension: Linda S. Pescatello, PhD
