# Supplementary material for: The “Hypertension Approaches in the Elderly: a Lifestyle study” multicenter, randomized trial (HAEL Study): rationale and methodological protocol
Source: BMC Public Health. 2019 May 29;19:657. doi: 10.1186/s12889-019-6970-3 (PMC6542055; doi:10.1186/s12889-019-6970-3)
Supplement: Supplementary file 2 — World Health Organization Trial Registration Dataset. (DOCX 13 kb) [file 12889_2019_6970_MOESM2_ESM.docx]

**Additional file 2**

**World Health Organization Trial Registration Dataset**

1. **Primary Registry and Trial Identifying Number**
   ClinicalTrials.gov, NCT03264443
2. **Date of Registration in Primary Registry**August 28th 2017
3. **Secondary Identifying Numbers**
   Federal IRB Number: CAAE 62427616.0.1001.5327

WebGPPG Number: 17-0044

1. **Source(s) of Monetary or Material Support**

CNPq (Conselho Nacional de Desenvolvimento Científico e Tecnológico)

FAPERGS (Fundação de Amparo à Pesquisa do Estado do Rio Grande do Sul)

CAPES (Coordenação de Aperfeiçoamento de Pessoal de Nível Superior)

FIPE (Fundo de Incentivo à Pesquisa e Eventos, Hospital de Clinicas de Porto Alegre)

1. **Primary Sponsor**
   Hospital de Clínicas de Porto Alegre
2. **Secondary Sponsor(s)**
   Federal University of Pelotas
3. **Contact for Public Queries**
   Daniel Umpierre, PhD
   Hospital de Clínicas de Porto Alegre
   Rua Ramiro Barcelos, 2350 - Hospital de Clínicas de Porto Alegre, Clinical Research Center, 21301
   Porto Alegre, RS, Brazil
   Ph: +55 51 3359.6332 / Fax: +55 51 3334.6462
   Email: daniel.umpierre@ufrgs.com
4. **Contact for Scientific Queries**
   Lucas Porto Santos, MSc
   Hospital de Clínicas de Porto Alegre
   Rua Ramiro Barcelos, 2350 - Hospital de Clínicas de Porto Alegre, Clinical Research Center, 21301
   Porto Alegre, RS, Brazil
   Ph: +55 51 3359.6332
   Email: lucaspsantos87@gmail.com
