# Supplementary material for: The “Hypertension Approaches in the Elderly: a Lifestyle study” multicenter, randomized trial (HAEL Study): rationale and methodological protocol
Source: BMC Public Health. 2019 May 29;19:657. doi: 10.1186/s12889-019-6970-3 (PMC6542055; doi:10.1186/s12889-019-6970-3)
Supplement: Supplementary file 3 — Amendments chronology. (DOCX 12 kb) [file 12889_2019_6970_MOESM3_ESM.docx]

**Additional file 3**

**Amendments chronology**

Protocol amendment number: 01

Authors: Lucas P. Santos, Daniel Umpierre

Issue date: 04 Out 2017

Primary reasons for amendment: inclusion of (1) an individual participant data sharing plan, and (2) a body composition imaging procedure in the protocol text and written informed consent.

Additional corrections: corrected phrasing for ultrasonography procedure, inclusion of periodic evaluations of blood pressure during the intervention sessions.

Protocol amendment number: 02

Authors: Cíntia E. Botton, Daniel Umpierre

Issue date: 23 Jan 2018

Primary reason for amendment: inclusion of a recruitment strategy in Section 4.2.1, which therefore includes access to a patient list originated by electronic medical records from the primary care facility within the Hospital de Clínicas de Porto Alegre.
